# Actinomycotic brain abscess

**DOI:** 10.1259/bjrcr.20150370

**Published:** 2016-11-02

**Authors:** Maryam Rahiminejad, Harutomo Hasegawa, Marios Papadopoulos, Andrew MacKinnon

**Affiliations:** ^1^Department of Neurosurgery, Atkinson Morley Wing, St George’s Hospital, London, UK; ^2^Department of Neuroradiology, Atkinson Morley Wing, St George’s Hospital, London, UK

## Abstract

Actinomycosis is caused by Gram-positive filamentous anaerobic organisms of genus *Actinomyces*, which are commensals of mucosal membranes of the oropharyngeal cavity, and gastrointestinal and genitourinary tracts. Central nervous system involvement is rare and may present as cerebral abscess, meningitis, meningoencephalitis, subdural empyema or epidural abscess. The radiological appearances of actinomycotic brain abscesses are not well recognized. Here, we present the characteristic imaging features of an actinomycotic brain abscess.

## Clinical presentation

A 50-year-old male presented with a 2-day history of progressive difficulties with his speech. He reported no other relevant symptoms. His past medical history included ischaemic heart disease and cardiac stents. He smoked 20 cigarettes per day. There was no history of immunosuppression. Neurological examination revealed an alert patient with mild expressive dysphasia and right-sided facial weakness. The remainder of the physical examination was normal. He was apyrexial and the pulse rate, blood pressure and oxygen saturations were normal. Blood tests, including full blood count, urea and electrolytes, and liver function tests were normal. The C-reactive protein level was 29.6 mg l^−1^ (normal range 0–10 mg l^−1^).

## Imaging findings

**Figure 1.**
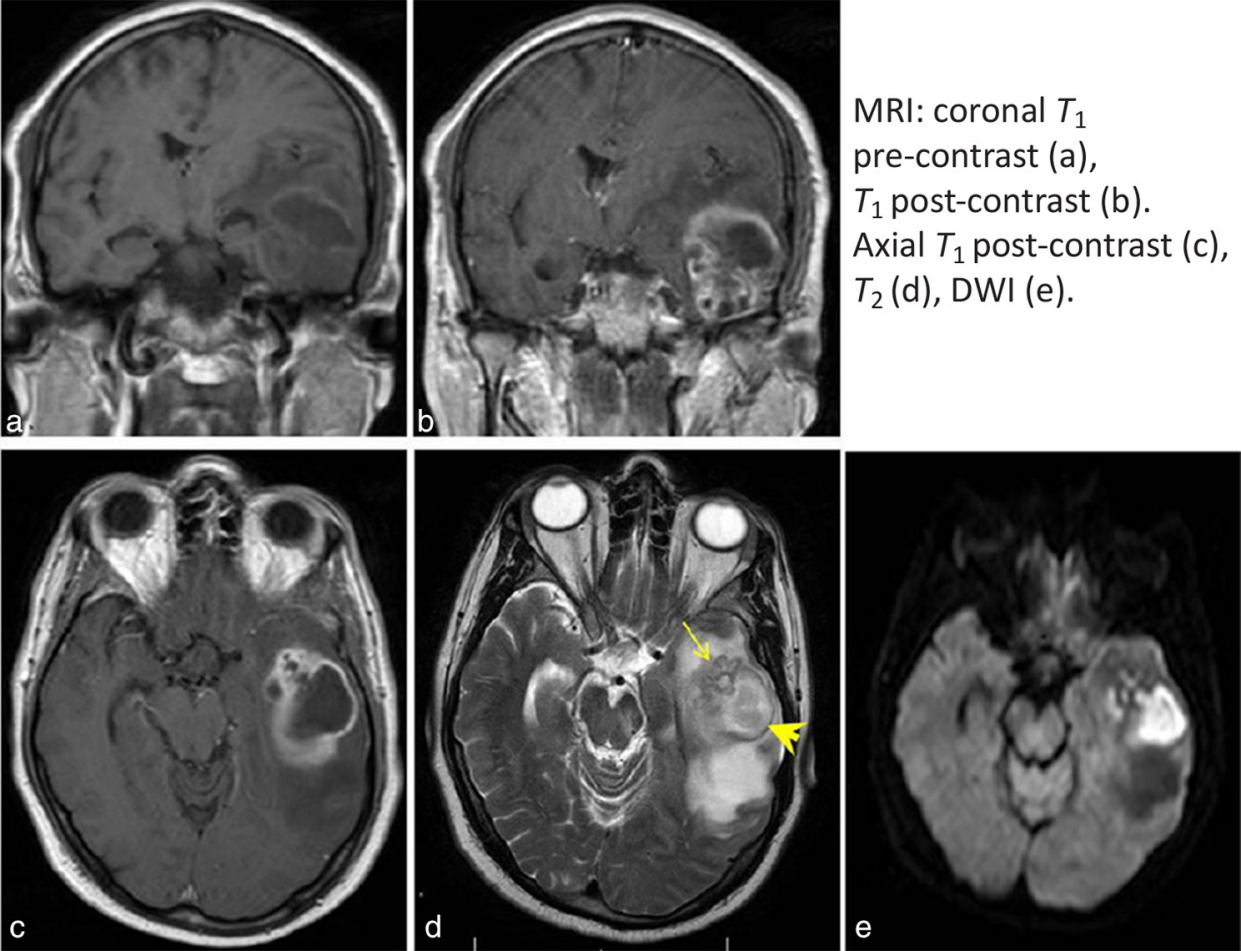
There is a large, peripherally enhancing thick-walled lesion in the left temporal lobe. The lesion comprises a larger cavity posteriorly and grape-like clustering anteriorly (arrow). The wall is *T*_2_ hypointense and *T*_1_ hyperintense (arrowhead). The contents of the lesion show restricted diffusion, which is consistent with an abscess. The diagnosis is suggested by the grape-like cluster pattern with a *T*_2_ hypointense wall, which is a characteristic feature of an actinomycotic abscess. DWI, diffusion-weighted imaging.

MRI demonstrated a large, peripherally enhancing thick-walled lesion in the left temporal lobe. The lesion comprised a larger cavity posteriorly and grape-like clustering anteriorly (Figure 1, arrow). The wall was *T*_2_ hypointense and *T*_1_ hyperintense (arrowhead) (Figure 1). The contents of the lesion showed restricted diffusion.

The principal differential diagnosis of a peripherally enhancing lesion in the brain is between a necrotic or cystic tumour and an abscess. Radiological diagnosis of the lesion is important because it may influence the initial surgical strategy. Tumours typically present insidiously, whereas a cerebral abscess may present rapidly with clinical features of infection. The history and clinical examination may not, however, be suggestive, especially in abscesses caused by atypical organisms. Radiologically, an irregular peripheral enhancement pattern can be seen with high-grade intrinsic tumours such as glioblastoma multiforme. Pyogenic abscess walls are usually smooth and well defined with a disproportionate amount of oedema. Homogeneous restricted diffusion in a smooth-walled enhancing lesion is suggestive of a pyogenic abscess, whereas restricted diffusion related to a high-grade tumour is often heterogeneous.^1^ Infections that may appear similar to brain tumours on imaging include *Actinomyces*, *Nocardia*, tuberculous granuloma, neurocysticercosis and eumycetoma.

## Treatment

The patient underwent stereotactic aspiration of the lesion and 16 ml of pus was aspirated. Cultures grew *Actinomyces meyeri* and *Fusobacterium nucleatum*. The treatment of actinomycotic brain abscess includes surgical aspiration and a substantive course of antibiotics.^2^ Our patient was treated with metronidazole and clindamycin following surgical aspiration. The abscess recurred after a month and required re-aspiration, but subsequently responded to further antibiotic treatment. We were unable to identify the source of infection in our patient, although periodontal disease, which is associated with smoking,^3^ may have played a role.

## Discussion

**Figure 2.**
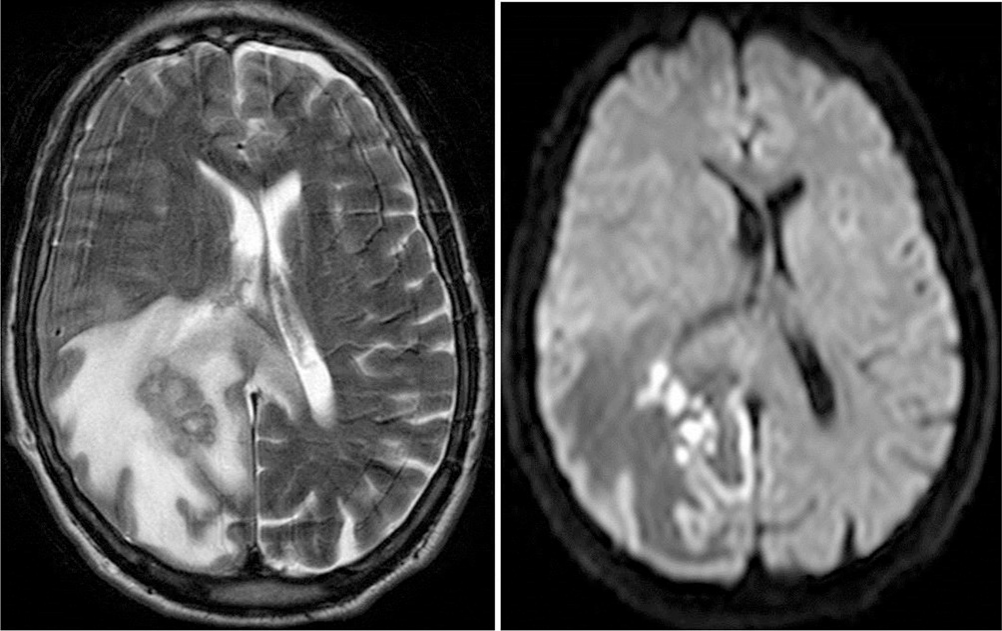
Axial *T*_2_ and diffusion-weighted MRI in a 60-year-old male with an actinomycotic brain abscess in the right parietal lobe with surrounding oedema. There is grape-like clustering with a *T*_2_ hypointense thick wall. The contents show restricted diffusion.

There is a paucity of literature with focus on the radiological appearances of actinomycotic brain abscesses.^4,5^ Imaging descriptions in case reports often do not contain much detail on characteristic radiological features.^6–8^ Actinomycotic brain abscesses have been described as irregular, thick and nodular,^5^ or thin^4,6^ peripherally enhancing lesions^4–9^ with a hypointense core and a rim that is hyperintense on *T*_1_ non-contrast imaging^4,5^ and hypointense on *T*_2_ images.^5^ Restricted diffusion of the core is often seen^4,5,7^ but is not the rule.^9^ The periphery may not restrict the diffusion.^4,5^ In our patient, the diagnosis was suggested by the grape-like cluster pattern with a *T*_2_ hypointense wall, which is a characteristic feature of an actinomycotic abscess, which, to our knowledge, has not been reported in the literature. This imaging appearance was also well demonstrated in another patient treated in our department, a 60-year-old male with no relevant medical history who presented to our department with confusion and falls, and was found to have an actinomycotic brain abscess (*A. meyeri*). MRI showed the *T*_2_ hypointense grape-like clustering pattern in the right parietal lobe (Figure 2).

Actinomycosis is caused by Gram-positive filamentous anaerobic organisms of genus *Actinomyces*, which are commensals of mucosal membranes of the oropharyngeal cavity, and the gastrointestinal and genitourinary tracts.^2,10^ *Actinomyces israelii* is most commonly isolated in clinical infections. *A. meyeri* is less common but has a propensity to cause disseminated disease.^2,11^ Infection begins with a breach of the mucosa and is associated with poor dental hygiene, trauma and intrauterine devices. Infection is often polymicrobial and can be associated with *Fusobacterium* (as in this case)^12^ or other commensal organisms.^2^ Brain abscesses represent approximately two-thirds of central nervous system infections, the rest being meningitis, encephalitis, subdural empyema and epidural abscess.^10^ The organisms grow in clusters of tangled filaments and may exhibit an outer zone of granulation around the central purulent fluid, which contains tiny yellow clumps (“sulfur granules”) formed by a matrix of bacteria, calcium phosphate and host tissue.^13^ The granulation zone is usually very thick and consists of a highly cellular fibrous tissue containing collagen fibres, fibroblasts, capillaries and inflammatory cells, mainly lymphocytes and monocytes.^14^ We postulate that these pathological features may lead to the grape-like clustering pattern, although it is interesting that this imaging feature has not been reported in *Nocardia* and fungal infections that share similar morphological and pathological features.^15^

## Learning points

1. Actinomycotic brain abscess is a rare but potentially life-threatening infection.
2. Actinomycotic brain abscesses appear on MRI as peripherally enhancing lesions that may exhibit a hyperintense rim on *T*_1_ non-contrast imaging and a grape-like cluster pattern with a *T*_2_ hypointense wall.

## Consent

We were unable to obtain signed informed consent from our patient as the patient is deceased and attempts to contact the next of kin were unsuccessful. Exhaustive attempts have been made to contact the family and the paper has been sufficiently anonymized not to cause harm to the patient or his family.
